# Cardiac rehabilitation in Canada and Arab countries: comparing availability and program characteristics

**DOI:** 10.1186/s12913-015-1183-7

**Published:** 2015-11-26

**Authors:** Karam I. Turk-Adawi, Carmen Terzic, Birna Bjarnason-Wehrens, Sherry L. Grace

**Affiliations:** School of Health Policy and Management, York University, Toronto, Canada; Department Physical Medicine and Rehabilitation, Mayo Clinic, Rochester, USA; Institute for Cardiology and Sports Medicine, German Sport University Cologne, Cologne, Germany; School of Kinesiology and Health Science, York University, 4700 Keele Street, Toronto, M3J 1P3 ON Canada; GoodLife Fitness Cardiovascular Rehabilitation Unit, University Health Network, Toronto, ON Canada

**Keywords:** Cardiac rehabilitation, Availability, Arab countries

## Abstract

**Background:**

Despite the high burden of cardiovascular diseases in Arab countries, little is known about cardiac rehabilitation (CR) delivery. This study assessed availability, and CR program characteristics in the Arab World, compared to Canada.

**Methods:**

A questionnaire incorporating items from 4 national / regional published CR program surveys was created for this cross-sectional study. The survey was emailed to all Arab CR program contacts that were identified through published studies, conference abstracts, a snowball sampling strategy, and other key informants from the 22 Arab countries. An online survey link was also emailed to all contacts in the Canadian Association of Cardiovascular Prevention and Rehabilitation directory. Descriptive statistics were used to describe all closed-ended items in the survey. All open-ended responses were coded using an interpretive-descriptive approach.

**Results:**

Eight programs were identified in Arab countries, of which 5 (62.5 %) participated; 128 programs were identified in Canada, of which 39 (30.5 %) participated. There was consistency in core components delivered in Arab countries and Canada; however, Arab programs more often delivered women-only classes. Lack of human resources was perceived as the greatest barrier to CR provision in all settings, with space also a barrier in Arab settings, and financial resources in Canada. The median number of patients served per program was 300 for Canada vs. 200 for Arab countries.

**Conclusion:**

Availability of CR programs in Arab countries is incredibly limited, despite the fact that most responses stemmed from high-income countries. Where available, CR programs in Arab countries appear to be delivered in a manner consistent with Canada.

## Background

Cardiovascular disease (CVD) accounts for 10 % of the global burden of disease [1]. It is the leading cause of mortality globally, with over 17 million deaths annually [1]. Over the past two decades, CVD death rates have increased at an alarming rate in low- and middle-income countries (LMICs) [2], with more than 80 % of CVD deaths globally occurring in these countries [1].

The burden of CVD is particularly high in Arab countries (i.e., where Arabic is the official language). Globally, this constitutes 22 countries, of which 3 (13.6 %) are LICs, 13 (59.1 %) are MICs, and 6 (27.1 %) are high-income countries (HICs) [3]. While data are not available by country [4], there are mortality data available on the Eastern Mediterranean Region (EMR) broadly, where 18 of the 22 countries are Arab [5], and the Middle East and North Africa (MENA) region, where 18 of the 21 countries are Arab [6–8]. These data show that 27 and 35 % of all deaths in the EMR and MENA regions respectively, are caused by CVD [9]. More specifically, of total deaths in the MENA region, CVD was the cause of 13 % in Somalia (lowest), up to 49 % of deaths in Oman. Moreover, this mortality burden is anticipated to increase to 60 % across the MENA region by 2020 [10]. CVD is also the leading cause of disability in these countries [11].

Contrary to HICs in the Arab world [12], CVD mortality rates have been declining in HICs in the Western world, due to advancements in medical technology and treatment [13]. For instance, in the HIC Canada, it is estimated that there are 70,000 myocardial infarctions each year, from which 77 % of patients survive [14]. Consequently, the number of people living with chronic CVDs and associated disability is increasing. Clearly, there is great need for secondary prevention in Arab countries and Canada.

Cardiac rehabilitation (CR) is an outpatient chronic disease management program delivering secondary prevention, in a cost-effective manner [15]. CR programs offer medical assessment, structured exercise training, patient and family education, and delivery of comprehensive management strategies to reduce and control CVD risk factors (e.g., hyperlipidemia, hypertension, obesity, physical inactivity, and smoking) [16]. Randomized controlled trials and meta-analyses have shown that participation in CR decreases morbidity and mortality by approximately 25 % when compared to usual care, and significantly reduces risk factors, improves health-related quality of life, and promotes a healthy lifestyle [17]. Even in the current era, CR participation is shown to result in better outcomes than routine clinical care [18]. CR can deliver equivalent, if not more, benefit to patients than medical therapies and percutaneous coronary intervention in low-risk patients [19]. There is some evidence to suggest CR programs are effective in the Arabic setting as well [20, 21].

Despite these benefits, a recent review demonstrated low availability of CR worldwide, with only 38.8 % of countries offering any CR. Importantly, 23 % of LMICs (8.3 % for LICs and 28.2 % for MICs), which have the greatest burden of CVD, offer this low-cost model of care, compared to 68.0 % of HICs [22]. More centrally, little is known about the characteristics of these programs, the components delivered, their capacity, as well as degree of patient access in LMICs. Several seminal surveys have been administered in high-income countries such as in North America (including Canada) and Europe, to capture this information [23–27]. Only 2 surveys have been carried out in LMICs, namely in Latin [28] and South America [29].

There have been no comprehensive studies that have explored CR delivery in other regions of the globe with a large burden of CVD, such as in Arab countries. Therefore, the objectives of this study were: (a) to explore the availability of CR programs in Arab countries; and (b) to describe and compare qualitatively the characteristics, components, delivery expenses, access, and barriers to CR programs in Arab countries to the HIC Canada. By incorporating a comparison group into the study, differences in CR delivery in Arab countries can be juxtaposed against established practice in the Western world.

## Methods

### Study design and procedure

This research was observational and cross-sectional in design. The study was approved by York University’s Office of Research Ethics (Toronto).

Identified contacts were sent an e-mail requesting their participation. CR staff in Arab countries were sent this email in Arabic to solicit their participation.

Informed consent was secured through an online consent form. The English-language survey was administered through a web-based program (Survey Monkey) to Canadian contacts, and was administered as an emailed document to Arab contacts. The survey was anonymous. Data were collected over 3 months in both the Arab countries (April-July, 2014) and in Canada (June-September, 2014).

Non-respondents were sent 3 follow-up e-mail reminders, at 2 week intervals, in English or Arabic as applicable. Finally, non-responding Canadian programs were also contacted by phone to enquire whether they received the survey email, or would like it re-sent to them.

### Sample

The sample consisted of CR programs in Canada and in countries where Arabic is the official language. The inclusion criterion was an outpatient CR program that offered: (1) assessment of risk factors for CVD, (2) supervised exercise, and (3) at least one other strategy to control CVD risk factors. Canadian CR programs were identifed from the Canadian Association of Cardiovascular Prevention and Rehabilitation directory [30].

The 22 Arab countries in the world are: Algeria, Bahrain, Comoros, Djibouti, Egypt, Eritrea, Iraq, Jordan, Kuwait, Lebanon, Libya, Mauritania, Oman, Palestine, Qatar, Saudi Arabia, Somalia, Sudan, Syria, Tunisia, United Arab Emirates, and Yemen [5]. CR programs in Arab countries were identified through several strategies. First, as per our previously-published review on global availability of CR [22], we contacted identified programs in Bahrain, Qatar and United Arab Emirates (HICs), as well as Algeria, Egypt and Tunisia (MICs). Second, with the help of an informational specialist, the first author updated the extensive search of: (1) MEDLINE, EMBASE, and Google Scholar for articles or abstracts on CR in these 22 countries; (2a) Internet search engines for “cardiac rehabilitation” in each country, and (b) CR services provided by hospitals within these countries. Finally, 2 authors (KA & SG) used a snowball sampling strategy via the International Council of Cardiovascular Prevention and Rehabilitation [31] and other key informants in cardiovascular associations / foundations or working in the Ministry of Health in Arab countries to identify programs.

### Measures

The first author initially undertook a literature search to identify studies reporting results of CR program surveys on a regional, national or greater, basis. The last author contacted key informants and identified authors to invite them to collaborate and share the original surveys. Corresponding authors of CR surveys administered in Canada [26], Latin and South America [29], Europe [32], and the United States [33] agreed to collaborate.

The South American CR survey was translated from Spanish to English, and reviewed carefully for quality of the translation. Some minor revisions were made to ensure the meaning of the items was as intended. The first and last author then combined content from all 4 surveys, deleting any redundant questions. The survey assessed: (1) general information about the CR program such as location (urban, rural, other), and whether it was hospital-based (yes, no); (2) characteristics of the CR program model (e.g., program duration, services offered, and linkage to community); (3) characteristics of the patient populations served in the CR program, and the average time to patient start of the program after hospital discharge; (4) program capacity, such as average number of patients/center/year; (5) information on reimbursement (i.e., hospital, patient, government, mixed) and program expenses; (6) CR human resources (e.g., available staff specialty); and (7) barriers to accessing the program and completion rates. The integrated survey was reviewed and approved by all authors.

### Statistical analyses

For the first objective, the presence of CR in each of the 22 Arab countries was described. For the second objective, descriptive statistics were used to describe all closed-ended items in the survey (i.e., means and standard deviations, or frequency and percent). All open-ended responses were coded using an interpretive-descriptive approach [34].

## Results

### Respondents

Eight CR programs were identified in Arab countries: 4 in Gulf countries (1 in Bahrain [HIC], 1 in Qatar [HIC], and 2 in United Arab Emirates [HIC]) and 4 in Africa (2 in Egypt [MIC] [35], 1 in Algeria [MIC], and 1 in Tunisia [MIC]). Five programs responded (62.5 % response rate): 1 in Bahrain, 1 in Egypt, 1 in Qatar, and 2 in United Arab Emirates. The CR programs in Tunisia and Algeria could not be reached because the email addresses of the corresponding authors listed in a conference abstract and a French-translated abstract, respectively, were invalid. With regard to Canadian CR programs, there were 128 unique programs in the directory validated through the emails and calls. Thirty-nine programs responded (30.5 % response rate).

Respondents from Arab countries were CR coordinators (*n* = 2), a manager, a supervisor and a cardiologist; whereas, respondents from Canada were most frequently CR coordinators (*n* = 14; 35.9 %), managers (*n* = 12; 30.8 %), and infrequently directors (*n* = 1; 2.6 %) and supervisors (*n* = 1; 2.6 %). Other (*n* = 11; 28.2 %) respondents were clinical nurse leaders, exercise therapists and physiotherapists.

### Program characteristics

CR program characteristics are presented in Table 1. CR program duration in Arab countries was <3 months. In Canada, 26 (66.6 %) respondents reported a program duration of 3–6 months, 5 (12.8 %) reported >12 months, 4 (10.3 %) reported <3 months and 4 (10.3 %) reported 7–12 months. Patients attended an average of 2.3 ± 1.5 (mean ± standard deviation) sessions per week in Arab countries and 2.3 ± 1.0 sessions per week in Canada. Only programs in Canada offered alternative delivery models, specifically 11 (28.2 %) offered community-based programs and 1 (2.6 %) home-based. However, 60 % of Arab programs offered women-only classes (Table 2).

**Table 1:** Characteristics of Cardiac Rehabilitations Programs

Characteristics	Arab		Canada	
	*N* = 5		*N* = 39	
	*n*	%	*n*	%
Geographic Setting				
Urban	4	80.0	25	64.1
Rural	1	20.0	9	23.1
Other	0	0.0	5	12.8
Program Funding Source				
Public	2	40.0	32	82.1
Private	2	40.0	4	10.3
Mixed	1	20.0	2	5.1
Other	0	0.0	1	2.6
Site				
Hospital	4	80.0	20	51.3
Inpatient cardiology service	4	100.0	15	75.0

**Table 2:** Assessment of Risk Factors and Components Offered

	Arab		Canadian	
	*n*	%	*n* ^a^	%
Risk Factors Assessed in the Program^c^				
Body Mass Index	5	100.0	35	100.0
Tobacco Use	5	100.0	35	97.2
Low and high-density lipoprotein	5	100.0	33	94.3
HbA1_c_ among patients with Diabetes	5	100.0	33	94.3
Total Cholesterol	5	100.0	32	94.1
Harmful Use of Alcohol	5	100.0	32	88.9
Blood Pressure	4	80.0	36	100.0
Waist Circumference	4	80.0	35	100.0
Triglycerides	4	80.0	27	81.8
Glucose for Non-Diabetics	4	80.0	27	81.8
Sleep Apnea	4	80.0	18	54.5
Time Being Sedentary	3	60.0	23	63.9
Other^d^	0	0.0	4	57.1
Cardiac Rehabilitation Components				
Patient Education	5	100.0	36	100.0
Initial Assessment	4	80.0	35	100.0
Exercise Prescription	4	80.0	35	100.0
Nutrition Counselling	4	80.0	34	100.0
Prescription or Titration of Secondary Prevention Medications	4	80.0	22	62.9
End of Program Re-Assessment	3	60.0	34	100.0
Supervised Exercise Training	3	60.0	35	100.0
Heart Rate Measurement	3	60.0	35	100.0
Communication with Primary Care	3	60.0	35	100.0
CVD Risk Factor Management	3	60.0	33	97.1
Relaxation techniques	3	60.0	33	97.1
Smoking Cessation	3	60.0	27	77.1
6-Minute Walk Test	3	60.0	24	72.7
Self-Management Training	3	60.0	22	66.7
Psychological Counselling	3	60.0	18	52.9
Women’s Only Classes	3	60.0	3	9.1
Physical Activity Counselling	2	40.0	35	100.0
Depression Screening	2	40.0	29	85.3
Exercise Stress Test	2	40.0	29	82.9
Other Stress Tests^e^	1	20.0	8	26.7
Alternative Forms of Exercise^b^	1	20.0	9	26.5

*Abbreviations*: *HbA1*_*c*_ Glycated Hemoglobin, *CVD* Cardiovascular disease

^a^Note: Some respondents did not answer each item and so valid percentages are reported

^b^e.g., yoga, dance, tai chi

^c^respondents were asked to check all that apply

^d^Other responses included: stress, depression, and arthritis (which is not a risk factor per se)

^e^For the Arab program, it was cardiopulmonary exercise test; for the Canadian programs, respondents reported nuclear stress test, stress echocardiogram, and cardiopulmonary exercise test

Risk factors assessed by CR programs are reported in Table 2. Risk factors assessed were highly consistent in both Arab countries and Canada, with blood pressure, body mass index, tobacco use, lipids, blood glucose, and harmful use of alcohol being most consistently assessed (≥80 %). CR programs in Arab countries did appear to assess for sleep apnea more often than Canadian programs.

CR components offered are also reported in Table 2. Patient education, initial assessment, exercise prescription and nutrition counselling were most consistently offered in both Arab and Canadian programs (≥80 %). However, physical activity counselling, supervised exercise training, communication with primary care, end of program re-assessment, depression screening, and exercise stress testing were reported less frequently in Arab than in Canadian programs. While caution is warranted due to the low number of programs in Arab countries, programs in these countries more often offered prescription or titration of secondary preventive medications, psychological counselling and women-only classes than programs in Canada. Having a link to exercise programs within the community was reported for only one (20 %) Arab CR program, yet was available in 25 (69.4 %) programs in Canada.

### CR healthcare providers and their training

In >50 % of programs both in Canada and Arab countries, physicians (cardiologists or specialists in internal medicine) held the overall responsibility for CR delivery (Table 3). Nurses were most routinely present during exercise sessions in Canadian and Arabic programs. Notably, there were fewer healthcare specialties represented in Arabic than in Canada’s CR programs (5 versus 10 specialties), with no psychologists, dietitians, kinesiologists or pharmacists on staff. In 4 (80.0 %) CR programs in the Arab countries, respondents reported their program staff had cardiopulmonary resuscitation training, versus 37 (94.9 %) programs in Canada.

**Table 3:** Type of Cardiac Rehabilitation Professionals

	Arab (*N* = 5)		Canadian (*N* = 39)	
	*n*	%	*n*	%
Profession with Overall Responsibility				
Cardiologist	3	60.0	10	26.3
Specialist in Internal Medicine	1	20.0	11	28.9
Nurse	1	20.0	4	10.5
Program Director/Manager	0	0.0	6	15.8
Exercise Physiologist	0	0.0	5	13.2
Consultant Physician	0	0.0	2	5.3
CR Professionals Routinely Present During Sessions				
Nurse	3	60.0	27	56.3
Cardiologist	2	40.0	2	4.2
Physiotherapist	1	20.0	17	35.4
Social worker	1	20.0	2	4.2
Exercise specialist	1	20.0	18	37.5
Other physician	0	0.0	5	10.4
Psychologist	0	0.0	1	2.1
Dietitian	0	0.0	6	12.4
Kinesiologist	0	0.0	17	35.4
Pharmacist	0	0.0	1	2.1

*Note*: Some respondents did not answer each item and so valid percentages are reported

All respondents from the Arab countries indicated the absence of any formal education or training programs for healthcare professionals regarding delivering CR services in their country. Several sources for formal education or training programs were reported by 16 Canadian respondents. The training programs reported were offered by the American College of Sports Medicine (*n* = 4), the Canadian Association of Cardiovascular Prevention and Rehabilitation, and by some universities and colleges.

When participants were asked to list educational materials (e.g., textbooks, guidelines) which were most used/useful for delivering CR in their country, only one (20.0 %) Arabic respondent listed a source, which was the Canadian CR Guidelines. Two (40.0 %) Arabic respondents and 37 (94.9 %) Canadian respondents stated that their regional Society / Association of Cardiology addresses the role of CR for the secondary prevention of CVD in its Position Statements or Guidelines.

### Patients entering CR

Respondents estimated that 31.2 ± 32.5 % of patients in Arab countries and 37.4 ± 26.6 % of patients in Canada had been screened for CVD risk factors prior to experiencing the cardiac event or procedure that led them to be referred to CR. They also estimated 21.1 ± 22.7 % of CR participants in the Arab CR programs, and 24.3 ± 26.6 % of CR participants in Canada’s, engaged in 150 minutes of moderate to vigorous-intensity physical activity each week prior to CR (i.e., guideline recommendation [16]). The average time to start a CR program following discharge from the hospital was estimated as 4.4 ± 4.2 weeks in Arab countries, and 5.8 ± 3.9 weeks in Canada.

### CR access and barriers

Respondent perceptions of barriers to CR participation are shown in Table 4. Lack of human resources and lack of space were equally perceived as the greatest barriers to participation in Arab countries. “Other” (*n* = 2) responses from Arabic respondents were lack of training and technical guidelines. In Canada, lack of financial resources, followed by lack of human resources, were perceived as the greatest barriers. “Other” (*n* = 2) responses from Canadian respondents were lack of patient awareness and lack of administrative support.

**Table 4:** Barriers to CR Participation, by Country

	Arab		Canadian	
	*N* = 5		*N* = 39	
Perceived Barriers to Greater Participation^a^	Mean	SD	Mean	SD
Lack of space	4.0	1.2	3.4	1.2
Lack of human resources	4.0	1.4	3.8	1.2
Lack of patient referral	3.8	1.8	3.2	1.4
Lack of financial resources/ budget	3.6	1.1	4.3	1.0
Lack of equipment	3.5	1.9	2.7	1.1
Patient Groups Perceived to Have Low Access^b^	*n*	%	*n*	%
Patients of low economic means	4	80.0	21	17.2
Patients with a disability	4	80.0	17	13.9
Women	4	80.0	12	9.8
Patients with musculoskeletal problems	4	80.0	11	9.0
Rural patients	2	40.0	24	19.7
Older patients	2	40.0	10	8.2
Everyone needs more access	NA	NA	9	7.4
Patients with lack of language proficiency of program	NA	NA	9	7.4
Minority ethnic groups	NA	NA	7	5.7
Patients of certain religious beliefs	NA	NA	2	1.6
Reasons for CR Non-completion (%)	Mean	SD	Mean	SD
Non-compliance	37.0	20.2	23.5	15.3
Return to work	33.8	30.9	23.6	15.2
Financial reasons	33.0	32.7	9.2	8.9
No longer medically necessary	20.0	21.6	4.4	6.5
Transportation barriers	5.5	5.3	21.3	17.4

*CR* cardiac rehabilitation, *SD* standard deviation, *NA* not available

^a^Mean program responses to patient participation on a 5-point Likert type scale from 1 (this is definitely not an issue) to 5 (this is definitely an issue)

^b^Respondents were asked to check all that apply

Subpopulations which were perceived to have less access to CR are shown in Table 4. Rural patients and those of low economic status were most often reported by Canadian respondents; patients of low economic status, with musculoskeletal conditions, disability, and women were most often reported by Arab respondents.

Respondents were asked to estimate the percentage of patients who leave the CR program prior to completion. Respondents in Arab countries estimated an average of 36.8 ± 14.9 %, while Canadian respondents estimated 22.3 ± 15.5 %. Perceived reasons for patient failure to complete CR programs are also presented in Table 4. Patient non-compliance followed by return-to-work were the most-often reported reasons by respondents in Arab countries and Canada. Financial reasons were also prominent in Arab countries, as were transportation barriers in Canada. Twelve (30.8 %) Canadian respondents reported another reason for non-completion, namely illness or medical issues; no Arabic respondents provided another reason.

### CR capacity

Respondents were asked to estimate the number of CR programs available in their country, where the 5 response options ranged from 0–20 to >500. Four (80 %) respondents from Arab countries estimated 0–20 programs. For Canada, 12 (30.8 %) respondents reported 101–250 programs, and 6 (15.4 %) respondents estimated 21–100 programs. For this and the below questions, other respondents selected “I do not know”.

Respondents were asked to estimate the number of patients that attended CR in their country in the last year, where the 5 response options ranged from 0–350 to >1000. More than half of respondents (*n* = 3; 60.0 %) in the Arab countries estimated that in 2013, 0–350 patients attended outpatient CR. About one-third of the Canadian respondents (*n* = 14; 35.9 %) estimated over 10,000 patients attended outpatient CR. Respondents were asked to estimate the percentage of all diagnostically-eligible patients that attended outpatient CR nationally in 2013, where the 5 response options ranged from 0–10 % to >60 %. For the Arab countries, 3 (60.0 %) respondents estimated 11–25 % of eligible patients, and 1 (20.0 %) respondent estimated 0–10 %. For Canada, 13 (33.3 %) respondents estimated 11–25 %, 11 (28.2 %) respondents estimated 26–40 %, 5 (12.8 %) were unsure, and the rest estimated > 41 % of the eligible patients.

Estimates of CR capacity are presented in Table 5. As shown, CR programs in Canada are serving more patients per year, yet less than they have capacity to serve. This situation is opposite in Arab countries, where they report treating more patients than they have the capacity to serve. In Arab countries, the mean number of patients/session served in CR programs was less than that of the Canadian CR, but the median was higher in Canada. Arab programs have a much lower staff-to-patient ratio than Canadian ones (1:1.5 vs. 1:5.0, respectively). All respondents (*n* = 5; 100 %) from CR programs in Arab countries reported that they have dedicated space for CR, whereas 31 (81.6 %) respondents from Canadian programs reported having such dedicated space.

**Table 5:** Cardiac Rehabilitation Capacity

	Arab countries		Canada	
	Mean ± SD	Median	Mean ± SD	Median
Capacity to serve (patients/year)	234.0 ± 175.2	180	588.5 ± 633.4	325
Patients served/year	224.2 ± 186.4	200	511.8 ± 557.5	300
Patients/year received funding to serve	150.0 ± 122.3	102	426.3 ± 516.8	237
Patients/session	5.4 ± 6.3	15	15.8 ± 11.2	10
Staff: Patient Ratio During Supervised Exercise				
Patient	2.2 ± 2.4	3	10.6 ± 8.2	10
Staff	1.4 ± 1.1	2	2.6 ± 5.5	2

*Abbreviation*: *SD* standard deviation

To increase CR program capacity, 2 (40 %) Arab respondents suggested having support from decision-makers, 1 (20 %) reported increasing program staffing, 1 (20 %) reported spending on equipment, and 1 (20 %) reported additional training. For Canada, of the 27 who responded to this open-ended question, 12 (44.4 %) reported greater staffing, 7 (25.9 %) reported additional space, 6 (22.2 %) reported additional funding, and 2 (7.4 %) suggested other resources.

### CR funding and expenses

Respondents were asked about reimbursement and funding models for their services. In Arab countries, 2 respondents (40 %) each reported the following type of payment for CR services: government, the patient, and private health insurance. In Canada, CR services were most often paid for by the hospital (*n* = 18, 48.6 %; which is funded by government), government (*n* = 17, 44.7 %), patients (*n* = 13, 34.2 %), and private health insurance (*n* = 4, 10.8 %),

Ratings of the expense associated with delivering CR are shown in Table 6. As shown, the most expensive elements reported by Arab respondents were exercise equipment, equipment / supplies for risk assessment, exercise stress testing, and front-line personnel. In Canada, the most expensive elements were front-line personnel, exercise stress testing, blood collection and lipid testing, followed by exercise equipment.

**Table 6:** Mean Perceived Expense (± standard deviation) Associated with CR Components^a^

	Arab	Canadian
Exercise Equipment	4.0 ± 1.7	3.3 ± 0.9
Exercise Stress Testing on Treadmill or Cycle Ergometer	3.6 ± 1.7	3.7 ± 1.5
Equipment/Supplies for Risk Assessment	3.6 ± 1.7	2.8 ± 0.9
Front Line Personnel	3.4 ± 1.5	4.2 ± 0.7
Space	3.0 ± 1.2	2.6 ± 1.4
Patient Education Materials	2.8 ± 1.1	2.5 ± 0.8
Blood Collection and Lipid Testing	2.6 ± 1.1	3.4 ± 2.0
Free Weights	2.6 ± 0.9	2.5 ± 1.0
Blood Pressure Assessment Device	2.4 ± 0.9	2.7 ± 1.1

^a^On a 5-point Likert type scale from 1 (free) to 5 (very expensive)

## Discussion

CR participation is related to significantly reduced mortality and morbidity [17], and therefore is recommended in cardiovascular practice guidelines around the world [36–38]. Given these benefits and its’ cost-effectiveness [15], it is disconcerting how little CR is available to those with CVD [22]. We identified only 8 CR programs in the Arab world, and many of these were newly opened. Only half (3/6) of HICs in the Arab world offer CR, compared to 68 % of HICs globally [22]. There is a gross misalignment between burden of disease / need and CR supply in the Arab world.

### Program characteristics

Considerable comparability in CVD risk factor assessment and CR components delivered by CR programs in Arab countries and Canada was observed. This could be explained by the similar economic status of these Arab countries (i.e., HICs), and therefore availability of resources. Moreover, the Arab personnel are developing their programs based on Western HICs such as Canada, reporting they base their delivery of CR on Canadian guidelines [39].

Some Arab CR program characteristics were also comparable to those of South America [29]. For example, the median number of patients served per year per program was 180 for South American and 200 for Arab countries, while it was 300 for Canada. Other similar findings observed in both Arab and South American countries were: >50 % of CR programs were located in hospitals; physicians were the most-frequently reported provider with overall CR program responsibility; and finally low rates of depression screening with 29 % in South American and 40 % in Arab programs, compared to 85 % in Canada.

Studies in Western HICs suggest that women prefer women-only CR classes/programs, and there is a growing trend toward implementation of such classes [32, 40]. In this study, just under 10 % of Canadian programs offered such classes, compared to 60 % of Arab programs. Indeed, availability of women-only classes in Arab countries is likely related to religious beliefs and cultural values around sex segregation, beyond certain family relationships, so women can exercise comfortably. Regardless of the women-specific classes, women in Arab countries remained a subpopulation with low access to CR. It is reported that about 50 % of women in the EMR are physically inactive and > 70 % of women in Bahrain, Kuwait, United Arab Emirates, and Saudi Arabia are obese [41]. Clearly there is need to provide services to this population, while being sensitive to cultural and religious mores. In studies in non-Arab HICs, family responsibility and lack of CR awareness are highlighted as barriers to CR among women [42]. Women’s CR barriers and strategies to overcome them must be explored in the Arabic context.

### Clinical and policy implications

There were no national CR guidelines or training programs identified in any of the Arab countries, of which 73 % are LMICs. Importantly, the only CR guidelines to our knowledge that have been developed for LMICs stem from Latin and South America [43] and China (personal communication, Dayi Hu, May 5, 2014). Moreover, HICs of the Arab world that have CR do not have country-specific CR guidelines. Delivery of CR programs in Arab countries is based on guidelines developed in Western HICs such as Canada. On the contrary, 57 % of European countries have national CR guidelines [32]. The World Heart Federation is promoting the development of region-specific guidelines for secondary prevention, to facilitate implementation. Accordingly, the International Council of Cardiovascular Prevention and Rehabilitation is currently developing guidelines for CR adaptation to low-resource settings.

Lack of human resources and dedicated space were equally reported as the largest barriers to CR in Arab countries, followed by lack of patient referral and financial resources/ budget. Similar barriers were reported in Latin and South America [28, 29]. Similarly, in HICs such as the United Kingdom [23] and in Canada [44], lack of human and financial resources, space, and patient referral, were identified as causes of low CR use. Accordingly, policy-makers should focus on CR infrastructure, resources, implementation, as well as promotion in the Arab World and Canada.

### Limitations

There were some limitations to this study. First, caution is warranted in generalizing the findings reported herein to Arab countries due to the small sample of 5 programs, as well as for Canada due to the low response rate to the survey. However, the response rate observed is between the rates reported in previous studies where CR programs were surveyed (i.e., United States 13.7 % [33] to Canada 84.4 % [26]). Second, again because of the small sample of 5 programs in the Arab world, quantitative comparison of program characteristics between Canadian and Arab countries was precluded. Third, despite the approach used to identify CR programs in Arab countries, there may be other programs we failed to capture. These programs might have different characteristics than those reported herein and therefore firm conclusions regarding the state of CR in Arab countries cannot be drawn. Fourth, capacity of CR programs was based on respondents’ estimates, hence they should also be interpreted cautiously.

## Conclusion

In conclusion, only 8 CR programs were identified in all 22 Arab countries. Programs in Arab countries and those in Canada address similar risk factors and offer similar components, however programs in the former less often provide exercise testing, depression screening and counselling. Perceived barriers to CR participation were comparable in the Arab World and Canada, with lack of CR infrastructure including human, physical, and financial resources primary. There is a need for CR guidelines tailored to the Arab setting, as well as significantly enhanced provision of training and CR services.

## Abbreviations

CR: cardiac rehabilitation
CVD: cardiovascular disease
EMR: Eastern Mediterranean Region.
HICs: high-income countries
LICs: low-income countries
LMICs: low- and middle-income countries
MENA: Middle East and North Africa region
MICs: middle-income countries
